# The time to address diagnostic needs in universal health coverage is now: Leveraging the scale up of national testing capacity for HIV viral load and SARS-CoV-2

**DOI:** 10.4102/ajlm.v11i1.1685

**Published:** 2022-06-22

**Authors:** Francesco Marinucci, Jens Dhein

**Affiliations:** 1 Abbott GmbH, Wiesbaden, Germany

**FIGURE 1:**
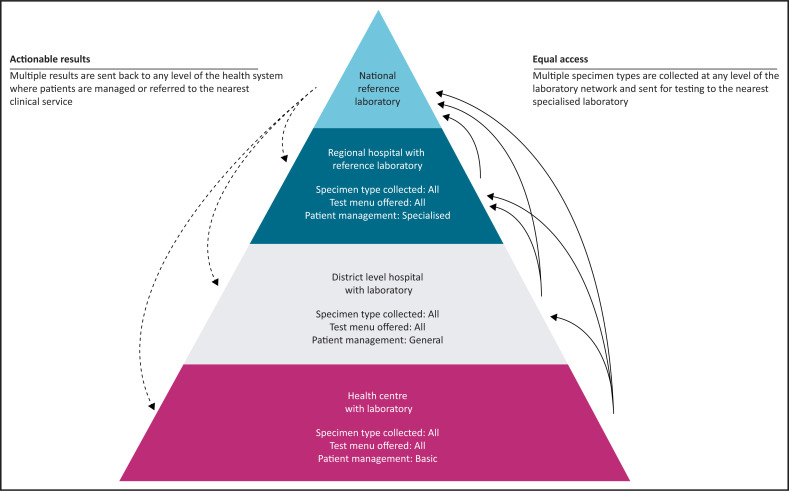
Vertical and horizontal integration of diagnostic services across the laboratory network allows all patients to access the same testing menu regardless of their entry point to the health system.

The goals of Universal Health Coverage (UHC) are to ensure that all people can access the quality health services that they need, to protect people from public health threats and to avoid catastrophic health expenditure.^1^ The range of essential services under UHC is country-specific and depends on factors such as the existing health system, the burden of different diseases and the resources available. As countries embark on defining their guaranteed package of services, the existing laboratory network represents an extraordinary opportunity to use the already existing equipment at its install base and full capacity across programmes, and to expand the laboratory testing menu in the UHC package. This article focuses on diagnostics and describes the reasons why flexible and high-throughput molecular platforms are critical to ensure access to quality testing and to improve diagnostic service coverage in the context of UHC initiatives. Integrated models of service delivery are instrumental for ensuring impactful and sustainable UHC interventions. Horizontal integration links healthcare professionals operating at the same level of care and ensures multiple diagnostic tests are available in the same laboratory tier. The focus of vertical integration is to increase coordination among testing sites and to improve communication within the different tiers of the laboratory network (Figure 1). As a result of the laboratory network optimisation, the laboratory system can fulfil the multiple needs of patients in more efficient and sustainable ways.^2^

Most testing has been carried out in central laboratories serving a large network of health facilities. Unfortunately, the role of laboratory-based platforms that allow the expansion of laboratories’ menus of assays (polyvalence) on the same analyser across different public health programmes has been undervalued, resulting in inadequate investments in vertical integration. This lack of investment has exacerbated the persistent issues related to communications between health facilities with the consequence of long turn-around times to get actionable results back to clinicians. Instead of strengthening the infrastructural gaps, the focus of different vertical programmes switched to bringing diagnostic services closer to patients, which increased the divides within laboratory networks.

The development of diagnostics that can generate results in primary healthcare settings has favoured the decentralisation of diagnostic services in several areas, including tuberculosis, HIV, viral hepatitis and malaria, with the main result being that diagnostic capacity is now closer to the patients.^3^ Global and national guidelines accompanied this transition to lower-level health facilities; however, many programmes for major diseases in low- and middle-income countries are still organised in vertical silos and lack the vision of combining platforms allowing menu expansion in different tiers of the laboratory network.^4^ Combined vertical and horizonal integration of diagnostic services across diseases has the potential to bring numerous benefits. For patients this results in more comprehensive care and services, added convenience due to time savings and reduced out-of-pocket expenses. For programmes, this results in cost savings from a more efficient use of resources, earlier treatment initiation and hence reallocation of resources to additional services. Lastly, this would increase the resilience of the health system to adapt to the onset of new diseases and public health threats that happen unexpectedly, such as outbreaks and epidemics.

**FIGURE 2:**
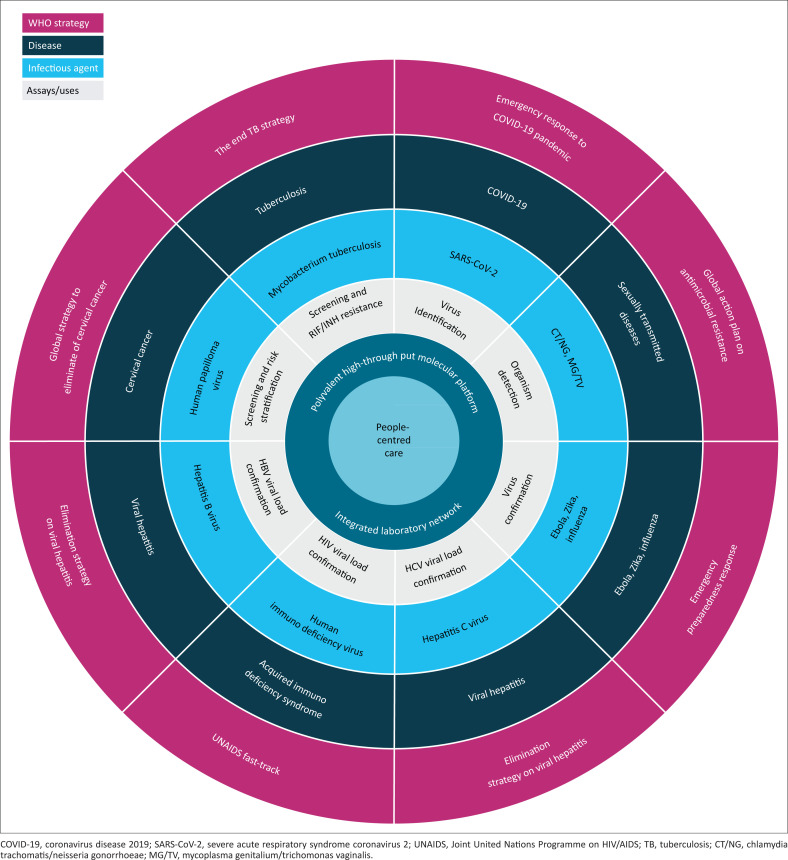
Link between global health strategies and automated molecular platforms.

The recent coronavirus disease 2019 (COVID-19) pandemic exacerbated the need for expanded diagnostic capacity in such situations and the importance of a quick response time to address the increased demand for testing.^5^ A significant number of countries, including low- and middle-income countries, have built up their infrastructure in a short time. Diagnostics manufacturers responded to the pressing need for COVID-19 testing with rapid development and the launch of new COVID-19 tests. The increased awareness of the importance of diagnostic testing provides the opportunity to look at other diseases that can be eliminated and where diagnostics plays a key role. Tuberculosis, HIV, cervical cancer and viral hepatitis, although treatable, are still underdiagnosed in many high burden countries. Considering these concerns, over the years the World Health Organization has released several global strategies to end these diseases^6^ (Figure 2). COVID-19 has demonstrated the advantages and limitations of current technologies to diagnose infections. The polymerase chain reaction is regarded as the gold standard for the detection of severe acute respiratory syndrome coronavirus 2 as a result of its high sensitivity for specific genetic sequences. Polymerase chain reaction-based techniques amplify this genetic material through multiple amplification cycles allowing the detection of small quantities of pathogens. Over the last 17 years many countries have scaled up their HIV viral load testing capacity with financial support from the United States President’s Emergency Plan for AIDS Relief and the Global Fund. The COVID-19 pandemic has pushed countries to further scale up their polymerase chain reaction testing capacities for severe acute respiratory syndrome coronavirus 2 by installing automated and high-throughput molecular diagnostic systems. Many of these systems have random access capability and allow the use of multiple polymerase chain reaction assays on the same platform. While demand for HIV viral load testing will remain stable in the near future, the demand for severe acute respiratory syndrome coronavirus 2 testing will likely decline, leaving molecular diagnostic testing platforms free and available for testing of other disease markers. With the equipment, reagents, skilled personnel, connectivity to the laboratory information system, training, and the technical support already in place, such automated platforms may help to achieve meeting the targets defined in the global strategies and UHC initiatives (Figure 2). Previous limitations would be mitigated since the increased level of automation reduces complexity and makes it easier for laboratories to perform testing. Random access systems eliminate the need for the sorting and batching of samples, provide continuous loading capability, and ready-to-use reagents. Larger on-board assay storage capacities and random access allow testing of low, medium and high-volume assays side-by-side. Testing capabilities could be rapidly adapted in response to increasing demand during a pandemic or in response to seasonal changes caused by respiratory viruses.^7^ Short sample-to-answer turn-around times allow same day reporting of results, with some of the molecular diagnostic platforms providing capability for sample prioritisation (true random access function) without disrupting routine sample processing.^8^ Menus comprising multiplex polymerase chain reaction assays allow differentiation of several pathogens in a single test, such as for respiratory or sexually transmitted infections. Multiplex assays enable laboratories to process more tests on demand in a given period of time. This conserves important testing materials that may be in short supply during an outbreak situation, and it saves sample volume that can be used for additional analyses. The addition of middleware solutions to the automated molecular diagnostic platforms could provide auto-verification capabilities that automate data transfer, improve turn-around time, reduce manual labour and the potential for human error. Centralised statistics and consolidated reports provided from the laboratories can give public health officials information for their ongoing surveillance programmes to control the spread of diseases and to monitor programme successes. Taken together, the HIV viral load and the COVID-19-driven uptake of molecular diagnostic platforms with expanded menus of assays provides a unique opportunity for laboratories to scale up molecular testing for HIV, viral hepatitis, tuberculosis, respiratory viruses, and high-risk human papillomavirus. The high automation level would enable laboratories to become more efficient in delivering high-quality results to support global health strategies in high burden countries. Furthermore, the flexibility of such platforms, both in terms of volumes and testing menus, place them at the forefront of regional initiatives for coordinated outbreak response and other initiatives of concern to public health.^9^ Simply increasing the number of shifts in the laboratory allows increases in the number of samples run on the platform. This flexibility is key for countries’ ability to rapidly cope with an increased demand for testing due to outbreaks and epidemics. The capability to use laboratory-developed tests on automated molecular diagnostic platforms can further strengthen the laboratories’ capabilities to respond in a timely manner to newly emerging diseases or variants.

While enabling scalability to react in an outbreak situation, an expanded menu of assays is a strong basis to continue expanding UHC packages. Countries can leverage existing sample referral networks, such as for HIV viral load testing, to serve multiple disease programmes by using the current infrastructure to move different specimen types from peripheral sites to the testing laboratory.^10^ The incremental cost-effectiveness of adding assays to the same high-throughput molecular diagnostic platform and the transportation of multiple specimens to the same laboratory would provide better use of scarce resources.^11^ This is particularly effective for infections like viral hepatitis C and human papillomavirus, where related diseases progress slowly, and access to results in a follow up visit does not impact clinical management. If required, results can be reported rapidly in situations where immediate clinical decisions must be made.^8^ Good coordination across multiple programmes is the foundation for more integrated testing to combine the advantages of both point-of-care tests and high-throughput molecular diagnostic platforms. Ideally, vertical and horizontal integration should occur simultaneously as part of the laboratory network optimisation supported by United States President’s Emergency Plan for AIDS Relief, the African Society for Laboratory Medicine and the Africa Centres for Disease Control and Prevention, with the ultimate goal of deploying laboratory-based platforms and point-of-care technologies where they are needed the most. High-throughput molecular diagnostic platforms, when used at full capacity, allow better use of resources across the laboratory network, which is central for achieving more people-centred care.
